# Endomyocardial Biopsy Revisited: Diagnostic Value and Expanding Roles in Cardiac Amyloidosis

**DOI:** 10.1007/s11897-026-00749-w

**Published:** 2026-03-13

**Authors:** Stéphanie K. Schwarting, Fabian aus dem Siepen

**Affiliations:** 1 Department of Medicine I, LMU University Hospital Munich, Marchioninistrasse 15, 81377 Munich, Germany; 2 Department of Cardiology, Angiology and Respiratory Medicine, University Hospital Heidelberg, Im Neuenheimer Feld 410, Heidelberg, Germany

**Keywords:** Endomyocardial biopsy, Cardiac amyloidosis, ATTR, Cardiomyopathy

## Abstract

**Purpose of Review:**

Cardiac amyloidosis (CA) results from myocardial infiltration by misfolded amyloidogenic proteins and represents a heterogeneous group of diseases with distinct prognostic and therapeutic implications. While transthyretin amyloidosis (ATTR) and immunoglobulin light-chain amyloidosis (AL) account for the majority of clinically relevant cases, several rarer amyloid subtypes with cardiac involvement have been described.This review focusses on the role of EMB in CA, highlights its diagnostic andprognostic value, discusses procedural considerations, and outlines emergingperspectives, including its potential role in phenotyping amyloid clearance andassessing treatment response in the era of novel disease-modifying therapies.

**Recent Findings:**

Over the past decade, diagnostic algorithms have shifted from predominantly invasive approach toward non-invasive imaging, particularly bone scintigraphy, which enables reliable diagnosis of ATTR in selected patients. However, important limitations remain. Non-invasive strategies may be insufficient in early disease stages, in the presence of monoclonal gammopathy, or in rare amyloid subtypes, where definitive amyloid typing is required. In these situations, EMB continues to play a central role. Additionally, EMB allows detailed assessment of amyloid burden and myocardial. Recent studies further demonstrate that beyond its diagnostic value, EMB also offers prognostic information, as higher amyloid load and inflammatory infiltration have been associated with adverse outcomes. Procedural risks appear acceptable when EMB is performed in experienced centers, although data on optimal biopsy strategies remain limited. In the era of emerging disease-modifying therapies, EMB may also gain an increasing relevance for disease phenotyping, assessment of amyloid clearance, and evaluation of treatment response.

**Summary:**

Despite major advances in non-invasive imaging, EMB provides the highest diagnostic accuracy in selected patients with suspected CA, enabling definitive amyloid typingand comprehensive myocardial tissue characterization that may contribute to refined disease phenotyping and the assessment of treatment response.

## Introduction

Amyloidosis is characterized by organ infiltration of misfolded amyloidogenic proteins. To date, more than 30 amyloidogenic proteins are known, but only a small subset of these shows a strong cardiac organotropism. The most clinically relevant forms are transthyretin (ATTR, including both hereditary and wild-type forms) and immunoglobulin light chains (AL), but rare cardiac involvement has also been described for serum amyloid A (AA), apolipoproteins (ApoA1/ApoA2), beta-2 microglobulin (Aß2M), fibrinogen, gelsolin and lysozyme [1–3].

Although these forms share a common final pathway - extracellular deposition of insoluble amyloid fibrils leading to disruption of myocardial contractility and electrical conduction – they significantly differ in terms of prognosis and therapeutic implications. Hence, precise and rapid subtyping of the amyloid form is crucial, which was recently only achieved by endomyocardial biopsy (EMB).

## Invasive Versus Non-invasive Diagnostic Approach to CA

**Fig. 1:**
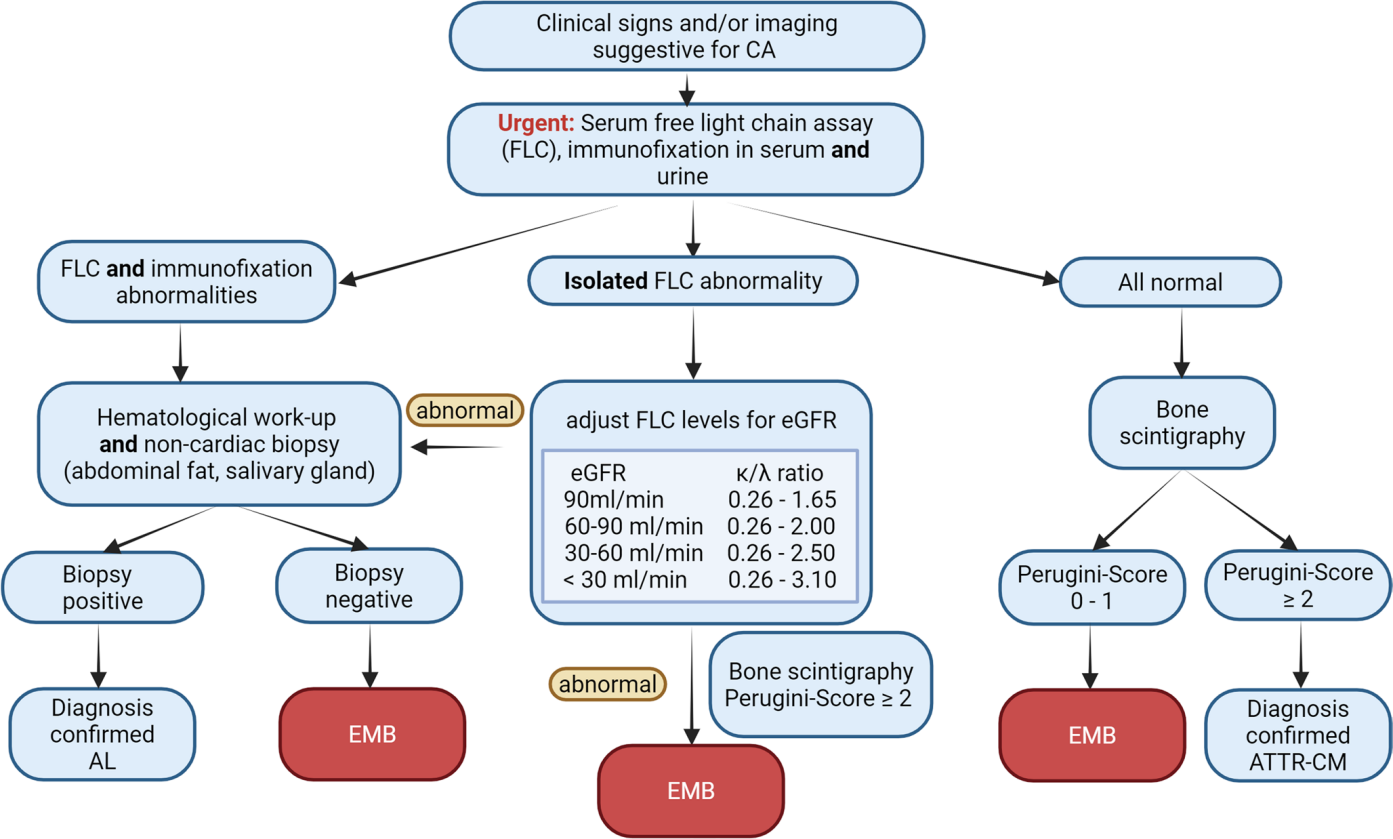
Diagnostic pathway to cardiac amyloidosis

Over the past decade, the diagnostic approach to cardiac amyloidosis (CA) has evolved from exclusively invasive to a predominantly non-invasive strategy. Bone scintigraphy with technetium-labeled bone-avid tracers (such as 99mTc-PYP, -DPD or -HMDP) has become a validated, guideline-endorsed, non-invasive diagnostic test for the ATTR subtype [1, 4, 5]. High diagnostic accuracy for ATTR is ensured only in high myocardial tracer uptake (Grade 2–3) after reliable exclusion of plasma cell dyscrasia (monoclonal gammopathy) (Fig. 1) [6]. Given the markedly different prognosis between ATTR and AL amyloidosis, rapid assessment of serum and urine free light chains together with immunofixation is essential and should be conducted at first sight. As the heart is the most frequently affected organ in AL amyloidosis, cardiac involvement – particularly its extent – represents the main determinant of survival [7]. Advanced disease is associated with a median survival of approximately four months if untreated [7]. Therefore, prompt exclusion of AL amyloidosis is crucial when applying diagnostic algorithms for suspected cardiac amyloidosis as it carries immediate and significant prognostic implications.

Flow chart outlining the diagnostic pathway, including myocardial biopsy, for the diagnosis of cardiac amyloidosis. (adapted from 2023 ACC Consensus [1]) (AL: light chain amyloidosis; ATTR-CM: transthyretin amyloid cardiomyopathy; CA: cardiac amyloidosis; EMB: Endomyocardial biopsy; eGFR: estimated glomerular filtration rate; FLC: free light chain).

Findings in bone scintigraphy, however, should be interpreted with caution, given the hypothesis that tracer uptake reflects both, a greater density of microcalcifications within ATTR deposits, and the underlying molecular fibril composition – type A (mixed fragmented and full-length) versus type B (full-length only) [8–11]. Additionally, some TTR variants (e.g. Ser77Tyr or Phe64Leu) have been reported to be associated with less or missing myocardial tracer uptake [12, 13]. Thus, a negative bone scintigraphy does not rule out CA and should prompt to pursue EMB to exclude false-negative results from scintigraphy and to assess for rare amyloid subtypes.

In order to establish definitive amyloid subtyping, indication for EMB in the context of CA is driven by the following scenarios: (1) strong and persistent clinical suspicion for cardiac involvement of systemic amyloidosis despite negative scintigraphy scan (Perugini grade 0–1), (2) positive scintigraphy findings (Perugini grade 2–3) in the concomitant presence of monoclonal gammopathy (accounting up to 25% of patients), (3) inconclusive or ambiguous imaging findings. In this context, the term “inconclusive” extends beyond Perugini grade 1 in scintigraphy. It also encompasses cases in which echocardiographic or cardiac magnetic resonance (CMR) findings show only subtle or incomplete features suggestive of cardiac amyloidosis, a scenario that is particularly relevant in early or subclinical disease stages. In such patients, typical, validated imaging criteria for amyloid cardiomyopathy may not yet be fully met, resulting in diagnostic uncertainty [5]. This is important because bone scintigraphy has primarily been validated in populations with higher pretest probability and is less established in low-probability settings such as in early disease stages.

**Fig. 2:**
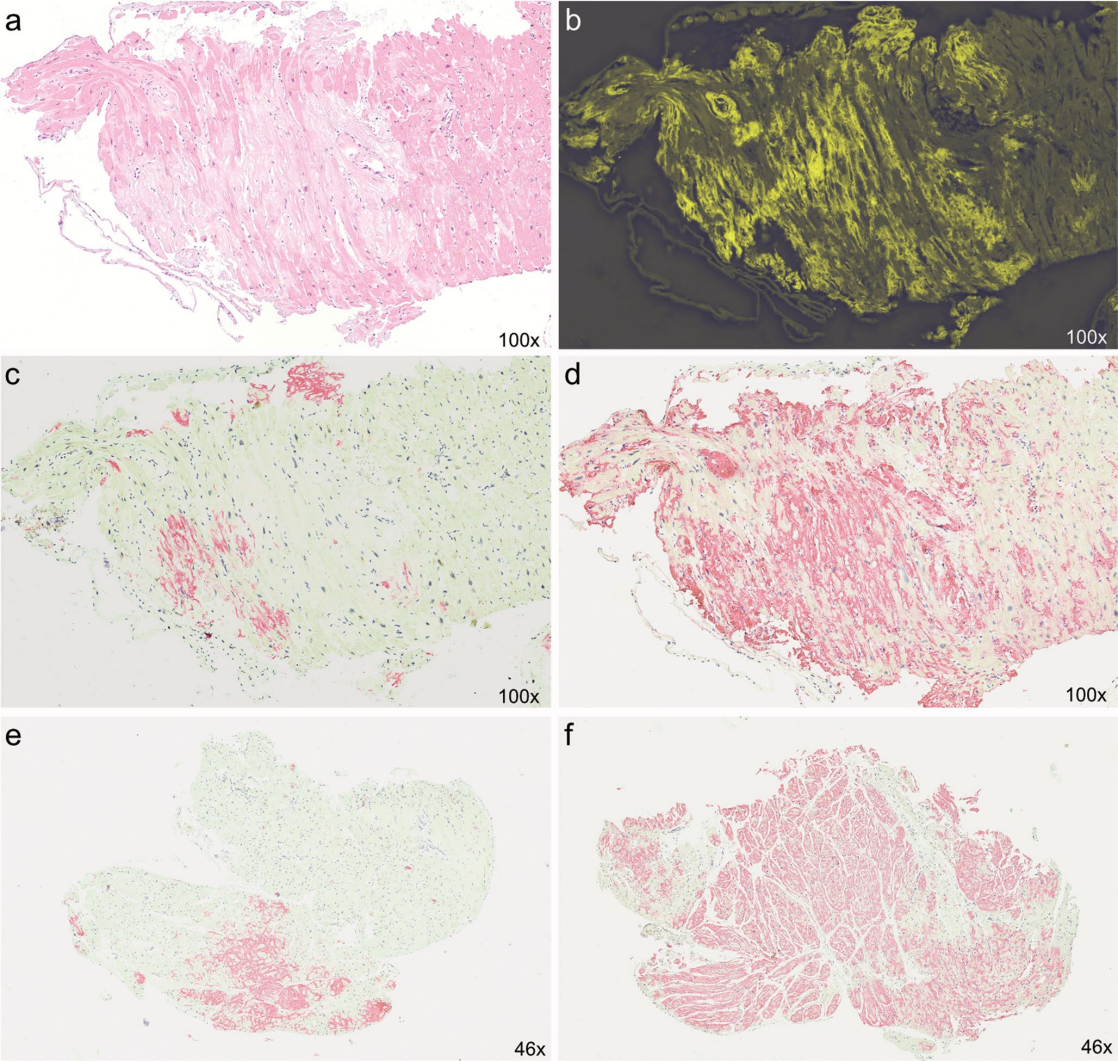
Histopathological subtyping of amyloid depositions in EMB

EMB has the highest diagnostic accuracy in CA and provides unique information on the amyloid subtype, quantity of amyloid infiltration, its distribution and concomitant inflammation. EMB samples will be screened for amyloid deposition in Congo Red staining and subsequent apple-green birefringence under polarized light. Detection rate of amyloid depositions can be further enhanced by electron microscopy [14]. Amyloid subtyping is achieved by immunohistochemistry or proteomic based typing methods, e.g. nano-liquid chromatography-tandem mass spectrometry analysis constituting the unique advantage of EMB over current imaging modalities, especially in the early disease stages, overlapping syndromes or inconclusive findings [15–18]. The myocardial distribution of amyloid deposits differs between AL and ATTR. AL amyloid predominantly shows a pericellular infiltration pattern, while ATTR amyloid tends to exhibit a patchy and diffuse interstitial distribution. Vascular wall involvement may accompany both forms [16]. Accurate differentiation of small or atypical amyloid deposits often necessitates analysis by a dedicated reference pathology center (Fig. 2).

The figure illustrates histopathological examples using different staining techniques for qualitative and quantitative assessment of amyloid deposition in a myocardial biosy. The first four panels depict the same biopsy specimen analysed with different stains, in which immunohistochemistry demonstrated a mixed form of amyloidosis with both AL (Panel d) and ATTR (Panel c) components (Panel a: haematoxylin and eosin (HE) staining, Panel b: congo red staining visualised by flourescence microscopy). Panel e & f: Quantification of amyloid burden, ATTR amyloid depositions involving 5.6% (panel e) and 18.0% (panel f) of the myocardial area. Original magnification x100 (Panel a-d) and x46 (Panel e-f). Images courtesy of Professor Christian Röcken, Kiel, Germany.

### EMB in Cardiac Amyloidosis – why?

The diagnostic yield of non-cardiac tissue biopsies - such as abdominal fat pad, rectal or salivary gland samples – varies depending on the amyloid type and the pattern of organ involvement [1]. Negative results from any of these “off-site” tissues do not exclude cardiac involvement, which is of paramount prognostic significance in any CA. Positive staining for ATTR in carpal tunnel tissue confirms systemic amyloidosis but does not necessarily indicate cardiac involvement and by itself, does not justify specific therapeutic intervention. Conversely, for AL amyloidosis or in cases of positive immunofixation, fat pad aspiration or other non-cardiac biopsy sites often provide sufficient diagnostic confirmation of systemic disease and already prompts immediate initiation of therapy. Cardiac involvement can help to decipher prognosis and guide further management in this subtype [19, 20]. Moreover, rare cases of mixed types of CA, e.g. AL and ATTR have been described, requiring complex therapeutic approaches (Fig. 2a-d).

## Performing EMB in CA – Considerations on Biopsy-related risk

EMB is an invasive procedure, thus certain aspects should be carefully considered beforehand. These include the choice of biopsy site – right versus left ventricular approach – as well as the potential risk of sampling error and the assessment of biopsy-associated risk for complications.

Biopsies obtained from both the left and right ventricle, particularly from the intraventricular septum, demonstrate comparable diagnostic yield in CA.^21^ The sampling error of EMB in CA is considered low, despite the focal and patchy myocardial distribution of amyloid deposits. Diagnostic sensitivity, however, increases substantially when more than four myocardial samples are obtained [16].

Fluoroscopy-guided EMB is considered safe if performed in a center with experienced interventionalists (Table 1) [22–24]. Periprocedural major complications include death, tamponade in need of pericardiocentesis, high grade, life-threatening ventricular arrhythmias, pacemaker dependency and ischemic stroke. In a general population, these occur in 0.3–10.7% with some evidence of greater risk in patients of older age with cardiomyopathy and somewhat dependent on localization of EMB [21, 24–28]. Minor complications such as small pericardial effusion, tricuspid valve injury, premature ventricular complexes or temporary atrioventricular block are observed more frequently than the major ones (about 3.3%- 20.7%).^23, 26, 28^

**Table 1:** Periprocedural risks associated with endomyocardial biopsy

Authors	Study Period	Study population and Biopsy Site	Bioptome Type	EMB-associated Complications	Cardiac Amyloidosis	Other findings
Yilmaz et al. 21	1995–2008	*N* = 755 (6,361 samples) LV: *n* = 265 (35.1%) RV: *n* = 133 (17.6%) Biventricular: *n* = 357 (47.3%)	Meiners Bioptome or Maslanka Bioptome	Major: LV 0.64% RV 0.82% Minor: total 3.8% LV 2.9% RV 5.1%	3.6%	Biventricular EMB yield higher diagnostic accuracy in myocarditis 2.500 IE heparin if LV-EMB
Holzmann et al. 22	1995–2005	*N* = 2,414 (3,048 samples) RV: 100%	6 F Cordis Bioptome (Meiners Germany)	Major: 0.12% Minor: 5.5%	Not reported	LBBB is more likely to lead to AV-block periprocedural
Chimenti et al. 24	1983–2010	*N* = 4,221 (6,617 samples) LV: *n* = 1144 (27.1%) RV: *n* = 672 (15.9%) Biventricular: *n* = 2,396 (56.8%)	King Bioptome BIPAL biopsy forceps (Cordis 7 F)	Overall: 0.39% LV 0.33% RV 0.45% biventricular: 0.66%	4%	Thinning of RV wall and age guide decision on site of EMB No differences of EMB site in diagnostic yield for CA 18% of LV hypertrophy were reclassified by results of EMB
Bermpreis et al. 28	2011–2021	*N* = 561 patients (1,368 samples; 914 repetitive for heart transplant) LV: *n* = 225 RV: *n* = 1,113	7 F Cordis Forceps	Overall: 4.1% Major: 0.8% Minor: 3.3%	4.7%	Higher rate of complications in cardiomyopathy patients (native hearts) and women (2.1% vs. 0.4% in male) No differences of EMB site in the rate of pericardial effusion or tamponade
Chatzantonis et al. 29	2016–2019	*N* = 160	NA	Overall: 3.13% Major: 2.5%	75%	
Kristen et al. 30	Not reported	*N* = 25	NA	Overall: LV 6.6% RV 17.1%	100%	
Singh et al. 44	2002–2013	*N* = 20,770 (71,105 samples)	NA	Major: 8.2% (Pericardiocentesis 2.6%, high grade AV block 2.7%, VT or cardiac arrest 2.8%	9.3%	
Vogelsberg et al. 45	2003–2006	*N* = 33	NA	No complications reported	45%	

Major complications: mortality, sustained VT (resuscitation needed), pericardial tamponade requiring pericardiocentesis, cerebrovascular event

Minor complications: AV-block in need of temporal pacing, non-sustained VT, transient hypotensive episodes, new pericardial effusion without need for pericardiocentesis

AV: atrioventricular; CA: cardiac amyloidosis; EMB: endomyocardial biopsy; NA: not available; LBBB: left bundle branch block; LV: left ventricle; RV: right ventricle; VT: ventricular tachycardia

Evidence on EMB-related complications in cardiac amyloidosis remains limited (Table 1). In recent studies, rate of EMB procedure-related complications in smaller cohorts of patients with suspected CA varied between 3.13% to 12.3%.^28–30^ However, neither study provided detailed characterization of risk-associated patient features that could inform procedural planning or the selection of the optimal biopsy site in CA. To date, there are no cardiac amyloidosis-specific studies offering clear guidance on the preferred location for EMB.

**Fig. 3:**
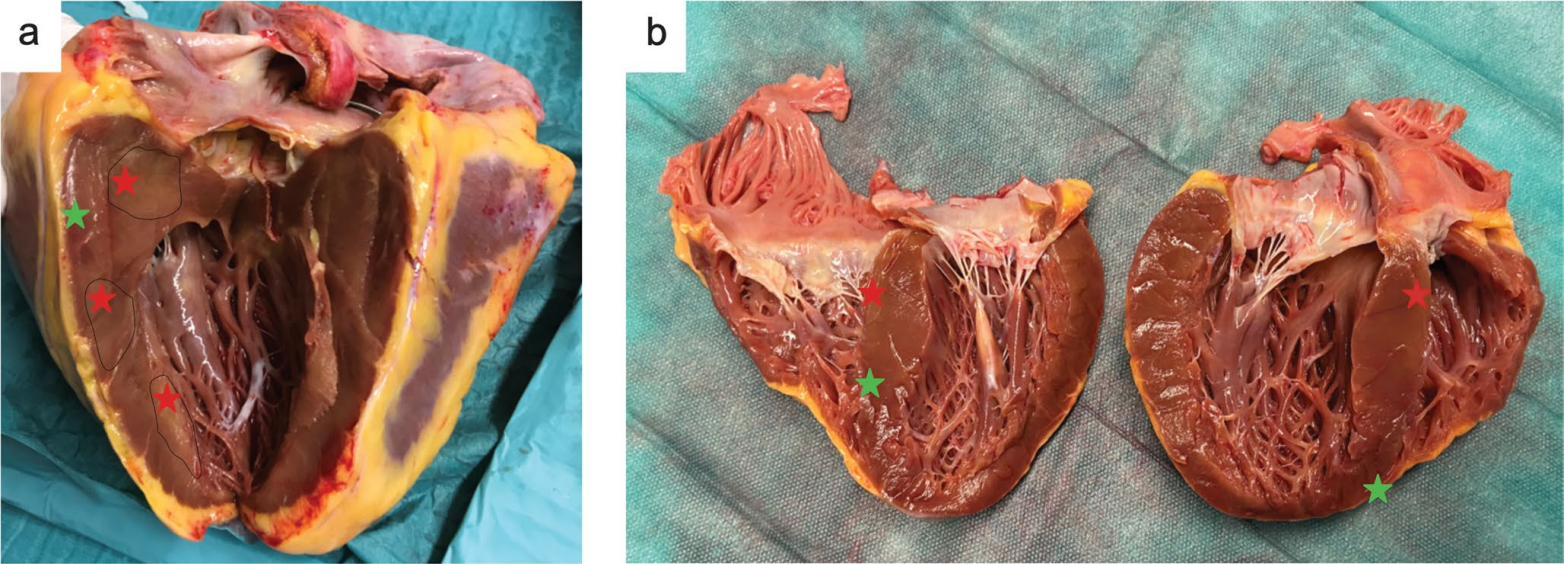
Macroscopic alterations in cardiac amyloidosis

In this context, two fundamental pathophysiological aspects of CA should be considered: presence of advanced conduction abnormalities and the paradoxical myocardial phenotype, characterized by increased wall thickness but pronounced tissue fragility and friability (“crumbly and spongy texture”). These features arise from fibril deposition and subsequent disruption of the extracellular matrix (Fig. 3).

Representative images of two explanted hearts from patients with cardiac amyloidosis undergoing heart transplantation (a: ATTR-CM, b: AL-CM). Both figures illustrate advanced myocardial involvement, with increased myocardial wall thickness and characteristic spongy appearance due to fibril depositions (red stars) in the extracellular matrix. Green stars denote undisrupted myocardium. (AL: light-chain amyloid cardiomyopathy; ATTR-CM: transthyretin amyloid cardiomyopathy).

To mitigate the risk of perforation or high-grade conduction disturbances, echo-guided biopsy sampling from the intraventricular septum may be considered, ideally employing an ECG-stratified approach to account for pre-existing conduction diseases such as complete bundle branch blocks. For instance, when a complete left bundle branch block (LBBB) is already present, LV EMB may be favoured, given that LBBB is more likely a sequela of CA rather than a procedure-related conduction problem. This concept could be further enhanced by using an electroanatomic voltage mapping (EAVM) to identify areas of risk such as the atrioventricular conduction system [31]. Additionally, EAVM also reveals potential to guide biopsy site reducing sampling error [32, 33]. To date, EAVM has not been widely adopted for procedural risk assessment, primarily due to the need for advanced equipment and the requirement of dedicated electrophysiological expertise.

Finally, the properties of the bioptome itself- including size, softness, and flexibility – and the experience of the interventionalist likely also play a role; however, these aspects have not yet been systematically investigated in any of the previous studies nor in the field of CA.

## Prognostic Implications of EMB in CA

Beyond its excellent diagnostic sensitivity, EMB in cardiac amyloidosis may also carry important prognostic implications.

Higher amyloid load in EMB samples (> 20%), reported as percentage of amyloid-positive pixels to total tissue pixels, typically correlates with late-stage disease and is associated with worse clinical outcome [34–36].

Moreover, presence of concomitant inflammation may carry important prognostic implications. Immunohistochemical analysis of EMB from patients with CA have demonstrated a frequent presence of CD68-positive macrophages in both AL and ATTR subtypes [8, 37, 38]. Despite inconsistent data on the association between these immune cells and amyloid burden, accumulating evidence suggests that resident innate macrophages play a role in local homeostatic regulation and may participate in both amyloidogenesis and amyloid clearance.

In AL-amyloid deposits, infiltration by CD3 + and LFA-1 + lymphocytes has been associated with adverse clinical outcomes [39]. In contrast, in ATTR-affected myocardial tissue, complement activation appears to play a more prominent role in the inflammatory milieu, particularly in patients with advanced disease stages. More recently, Muller et al. demonstrated that myocardial inflammation was present in approximately one third of EMBs from patients with ATTR-CM and, after adjustment for age and sex, was associated with a significantly increased risk of all-cause mortality and heart failure-related hospitalizations over an 18-month follow-up period [38]. Whether this immune-mediated response reflects a deleterious pathological process or represents an active, potentially compensatory clearance mechanism remains unclear. However, with respect to emerging therapies this aspect is of special interest. In fact, anti-amyloid fibril antibody-mediated enhanced macrophage activation and phagocytosis is the cornerstone of immunotherapeutic approaches, currently under investigation in clinical trials [40–42]. These depleting substances are thought to enable amyloid removal and consequently restore cardiac function.

In the absence of robust preclinical animal models, EMB tissue may facilitate the identification of a distinct phenotypic “fingerprint” indicative of endogenous amyloid clearance mechanisms [43]. This concept may be further advanced through single-cell and cell-matrix interaction analyses, which hold the potential to provide mechanistic insights into the early phases of disease development. In the era of emerging therapeutic options, a definitive assessment of treatment response will likely require serial EMBs; however, whether the anticipated scientific and clinical insights outweigh the associated procedural risks remain matter of debate.

Finally, recent advances in disease-modifying therapies for ATTR-CM, including stabilizer, RNA-targeting therapies and emerging amyloid-depleting approaches, have fundamentally reshaped the clinical management of the disease. These developments underscore the importance of early diagnosis, as treatment initiation at earlier disease stages may slow disease progression long before overt heart failure develops. Conventional imaging modalities may be less sensitive in early ATTR-CM, when structural and functional changes remain subtle. Currently, EMB is primarily reserved for diagnostically uncertain or inconclusive cases; with the increasing availability of effective therapies, EMB may assume a greater role in the early diagnostic confirmation of cardiac amyloidosis and may offer a favorable risk-benefit profile and enables earlier therapeutic intervention.

## Conclusion

Despite major advances in non-invasive imaging, endomyocardial biopsy remains a cornerstone in the comprehensive evaluation of cardiac amyloidosis. EMB continues to be indispensable in scenarios of diagnostic uncertainty, rare amyloid subtypes, early disease stages and in the presence of monoclonal gammopathy. Beyond definitive amyloid subtyping, EMB uniquely enables quantification of amyloid burden and characterization of associated inflammatory processes, all of which have relevant prognostic implications.

EMB carries an acceptable risk profile if performed at experienced centers. However, disease-specific features such as advanced conduction abnormalities and myocardial tissue fragility require careful procedural planning. Evidence guiding optimal biopsy strategies in cardiac amyloidosis remains limited and warrants further investigation.

In the context of emerging disease-modifying and immunotherapeutic strategies, EMB may have an expanded role beyond diagnosis by enabling phenotypic assessment of amyloid clearance and promises to deepen mechanistic insights to refine precision medicine approaches in cardiac amyloidosis.

## Data Availability

No datasets were generated or analysed during the current study.
